# Lu/BCAM Adhesion Glycoprotein Is a Receptor for *Escherichia coli* Cytotoxic Necrotizing Factor 1 (CNF1)

**DOI:** 10.1371/journal.ppat.1003884

**Published:** 2014-01-16

**Authors:** Marianne Piteau, Panagiotis Papatheodorou, Carsten Schwan, Andreas Schlosser, Klaus Aktories, Gudula Schmidt

**Affiliations:** 1 Institute for Experimental and Clinical Pharmacology and Toxicology, Albert-Ludwigs-University of Freiburg, Freiburg, Germany; 2 Biological Faculty, Albert-Ludwigs-University of Freiburg, Freiburg, Germany; 3 Rudolf-Virchow-Zentrum für Experimentelle Biomedizin, Universität Würzburg, Würzburg, Germany; 4 BIOSS (Centre for Biological Signalling Studies), Freiburg, Germany; University of California Los Angeles, United States of America

## Abstract

The Cytotoxic Necrotizing Factor 1 (CNF1) is a protein toxin which is a major virulence factor of pathogenic *Escherichia coli* strains. Here, we identified the Lutheran (Lu) adhesion glycoprotein/basal cell adhesion molecule (BCAM) as cellular receptor for CNF1 by co-precipitation of cell surface molecules with tagged toxin. The CNF1-Lu/BCAM interaction was verified by direct protein-protein interaction analysis and competition studies. These studies revealed amino acids 720 to 1014 of CNF1 as the binding site for Lu/BCAM. We suggest two cell interaction sites in CNF1: first the N-terminus, which binds to p37LRP as postulated before. Binding of CNF1 to p37LRP seems to be crucial for the toxin's action. However, it is not sufficient for the binding of CNF1 to the cell surface. A region directly adjacent to the catalytic domain is a high affinity interaction site for Lu/BCAM. We found Lu/BCAM to be essential for the binding of CNF1 to cells. Cells deficient in Lu/BCAM but expressing p37LRP could not bind labeled CNF1. Therefore, we conclude that LRP and Lu/BCAM are both required for toxin action but with different functions.

## Author Summary

We study a crucial virulence factor produced by pathogenic *Escherichia coli* strains, the Cytotoxic Necrotizing Factor 1 (CNF1). More than 80% of urinary tract infections (UTIs), which are counted among the most common bacterial infections of humans, are caused by Uropathogenic Escherichia coli (UPEC) strains. We and others elucidated the molecular mechanism of the *E. coli* toxin CNF1. It constitutively activates Rho GTPases by a direct covalent modification. The toxin enters mammalian cells by receptor-mediated endocytosis. Here, we identified the protein receptor for CNF1 by co-precipitation of cell surface molecules with the tagged toxin and subsequent Maldi-TOF analysis. We identified the Lutheran (Lu) adhesion glycoprotein/basal cell adhesion molecule (BCAM) as receptor for CNF1 and located its interaction site to the C-terminal part of the toxin. We performed direct protein-protein interaction analysis and competition studies. Moreover, cells deficient in Lu/BCAM could not bind labeled CNF1. The identification of a toxin's cellular receptor and receptor binding region is an important task for understanding the pathogenic function of the toxin and, moreover, to make the toxin accessible for its use as a cellbiological and pharmacological tool, for example for the generation of immunotoxins.

## Introduction

Urinary tract infections (UTIs) are among the most common bacterial infections of humans. More than 80% of UTIs are caused by Uropathogenic *Escherichia coli* (UPEC) strains [1]. Many pathogenic *Escherichia coli* strains including UPEC and strains inducing meningitis or soft tissue infections produce Cytotoxic Necrotizing Factor 1 (CNF1), a protein toxin which contributes to virulence [2]. Of major importance for its role as a virulence factor is the effect of CNF1 on epithelial barrier- and immune cell functions [3]. Both features are controlled by Rho GTPases, which are directly targeted by the toxin. CNF1 deamidates a specific glutamine (Gln63/61) of Rho proteins, which is crucial for GTP hydrolysis and therefore, the Rho proteins are arrested in a constitutively activated state [4], [5]. Rho family GTPases are regulated in a GTPase cycle by the following cellular proteins: GEFs (*guanine nucleotide exchange factors*) activate Rho proteins by GDP/GTP-exchange, GAPs (*GTPase-activating proteins*) stimulate the intrinsic GTP hydrolysis, thereby accelerating inactivation. GDIs (*guanine nucleotide dissociation inhibitors*) predominantly bind the inactive form of Rho GTPases blocking nucleotide exchange [6]. Active Rho proteins interact with several effectors, which govern a variety of cellular processes including organization of the actin cytoskeleton. Consequently, cells treated with CNF1 show a strong network of actin stress fibers, filopodia and membrane ruffles [7].

CNF1 belongs to a family of single chain AB-toxins with an N-terminal domain important for membrane translocation and the C-terminal catalytic domain encompassing deamidase activity. The toxin is taken up into mammalian cells by receptor-mediated endocytosis. It has been shown that the cytosolic precursor protein (37LRP) of the non-integrin laminin receptor (67LR) interacts with the N-terminus (aa 1–342) of CNF1 in a *yeast-two-hybrid*-screen [8]. Processing of 37LRP to the mature, membrane localized 67LR is still unclear but may originate from homo- or hetero-dimerization of 37LRP with another 37LRP molecule or with galectin3 [9]. Moreover, it has been suggested by knockdown of 37LRP that 67LR mediates the uptake of CNF1 into cells [8].

Here, we identified the Lutheran (Lu) adhesion glycoprotein/basal cell adhesion molecule (BCAM) as a cellular receptor for CNF1 and show that it is essential for the intoxication of cells. The transmembrane adhesion molecule Lu/BCAM is a member of the immunoglobulin (Ig) superfamily with five Ig-like domains on the extracellular site, a single transmembrane domain and a short, C-terminal cytoplasmic tail. This protein, like 67LR is a receptor for the extracellular matrix protein laminin. Two transcript variants, encoding different isoforms, have been found for this gene called Lu and BCAM. The difference between Lu and BCAM is the length of the cytoplasmic tail (59 amino acids in Lu and 19 amino acids in BCAM), which is thought to mediate intracellular signaling because it contains an SH3 binding motif and 5 phosphorylation sites [10]. Lu/BCAM binds to laminin α5, which is the major laminin α-chain in the basement membrane [11]. In humans, Lu plays a role in vaso-occlusion of red blood cells (RBCs) in sickle cell patients. In sickle cell RBCs, epinephrine increases a Lu/BCAM-mediated adhesion of the cells to laminin α5 in a cAMP and protein kinase A-dependent manner [12]. However, Lu/BCAM is also expressed in many other cells and tissues with a strong appearance in epithelial and endothelial cells (for review see [13], [14]). We show that CNF1 binds to Lu/BCAM on mammalian cells and that this interaction is crucial for the uptake of the toxin. The interaction of CNF1 with Lu/BCAM occurs within a region located in close proximity to the C-terminal catalytic domain, whereas the N-terminus of CNF1 interacts with the 37LRP precursor of the non-integrin laminin receptor [8]. Therefore, CNF1 seems to contact two different laminin binding proteins, using separate domains.

## Results

### Identification of Lu/BCAM as receptor for CNF1

Identification of the cellular receptor is a crucial task in understanding bacterial toxins. For CNF1 it has been reported that the N-terminal 342 amino acids of the toxin interact with the 37 kDa laminin receptor precursor (37LRP) [8]. This intracellular protein matures to the 67 kDa cell surface-localized laminin receptor (67LR). We confirmed a week interaction of CNF1 with 67LR by overlay assays (not shown). Therefore, we used purified 67LR from cells, which secrete this receptor into the culture medium [15]. However, some cell lines like RBL (rat basophil leukemia) or HeLaS3 (human cervix carcinoma, subclone 3) cells express 37LRP but are not intoxicated by CNF1, suggesting that another structure on the cell surface may be necessary for efficient binding and endocytosis of the toxin. In line with this, it has been shown, using monoclonal antibodies, that besides the N-terminal receptor binding domain, a second part of CNF1 is involved in binding to mammalian cells [16].

We screened for cell surface protein receptors CNF1 binds to by incubating HeLa cells with a double-tagged GST-CNF1-GST fusion protein at 4°C. As controls we used GST alone or the analogue double-tagged form of the CNF family member CNFY (GST-CNFY-GST, *Yersinia pseudotuberculosis* toxin CNFY). This toxin is known to interact with a different, yet unknown receptor on mammalian cells [17]. Following binding, we lysed the cells and precipitated the toxin together with associated molecules, using anti-GST magnetic beads. Eluates were separated on SDS-PAGE and the eluted proteins were subsequently identified by nanoLC-MS/MS. The only hit unique to the CNF1-precipitate was the Lutheran (Lu) adhesion glycoprotein/basal cell adhesion molecule (BCAM) (Fig. S1). This surface protein has a large extracellular Ig-like structure and is widely expressed. Interestingly, Lu/BCAM like the proposed CNF1 receptor 67LR interacts with laminin, suggesting that the receptor-binding domain of CNF1 could interact with both laminin binding structures on the cell surface.

**Figure 1:**
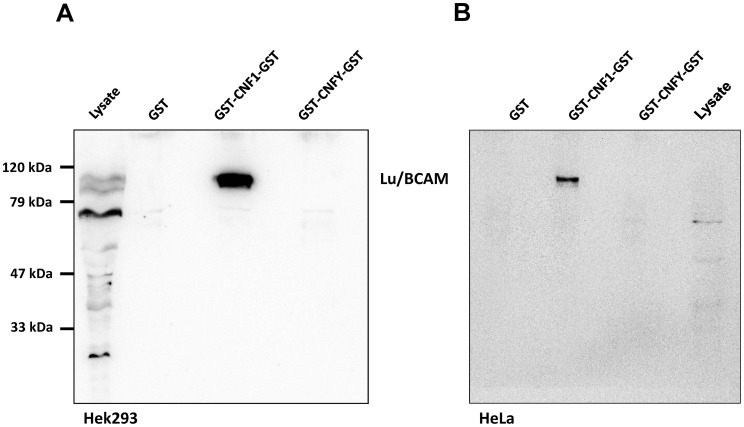
Lu/BCAM is co-precipitated with CNF1 but not with CNFY. HEK293 (A) or HeLa (B) cells were incubated with GST, GST-CNF1-GST or GST-CNFY-GST as indicated for 20 min at 4°C. Cells were harvested and lysed and the GST proteins together with their bound interaction partners were pulled with anti-GST magnetic beads. Proteins were separated by SDS-PAGE and co-precipitated Lu/BCAM detected by Western-blotting. Note that the major band for Lu/BCAM in the lysate runs at about 67-kDa. The glycosylated, surface exposed protein runs at higher molecular weight. Shown is an example of at least 3 independent experiments.

To verify the CNF1-Lu/BCAM interaction, we repeated the precipitation assay with HEK293 (Fig. 1A) and HeLa cells (Fig. 1B) and analyzed the presence of Lu/BCAM in the precipitate by Western-blotting with a specific antibody against Lu/BCAM. As shown in Fig. 1, Lu/BCAM was exclusively co-precipitated with GST-CNF1-GST but not with GST-CNFY-GST or GST alone. Notably, we could not detect 37LRP/67LR in any lane by Western-blotting although the protein was expressed in HeLa and in HEK293 (human embryonic kidney) cells (Fig. S2).

**Figure 2:**
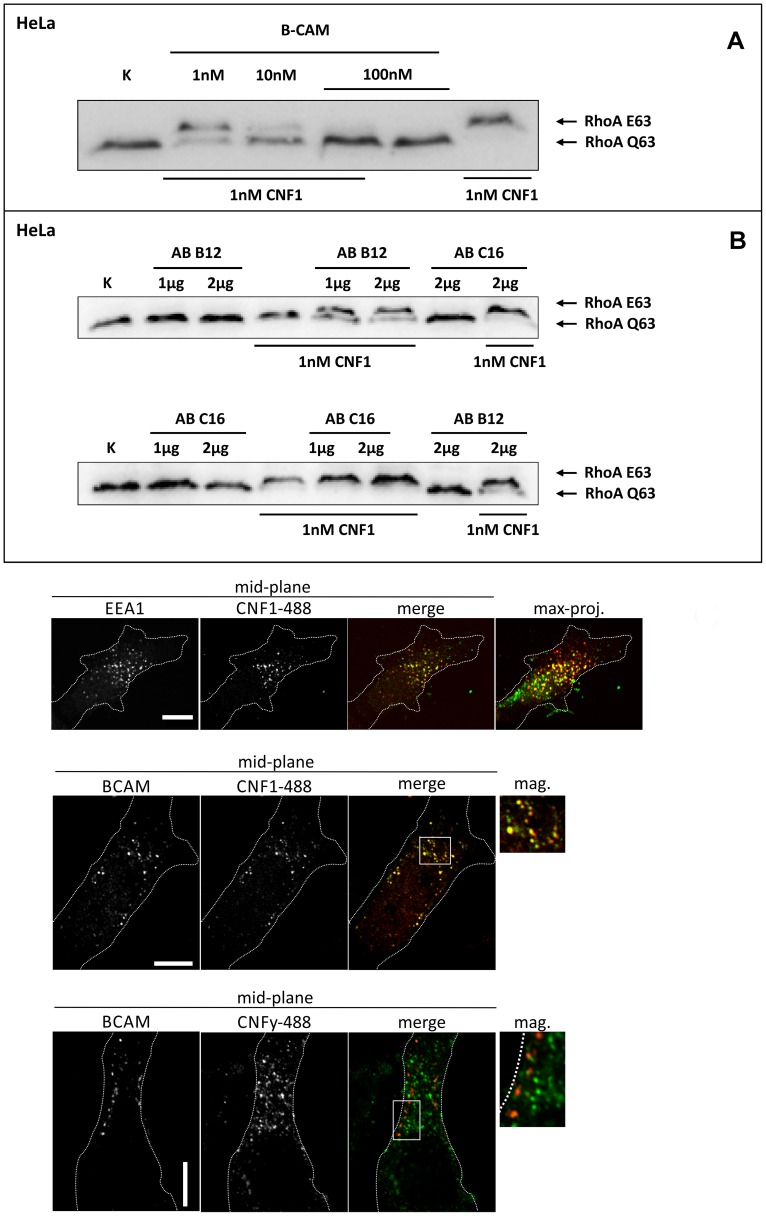
Competition and colocalization studies with HeLa cells. GST-CNF1 was incubated with buffer or with recombinant BCAM in a molar ratio CNF1∶rBCAM of 1∶1, 1∶10 and 1∶100, respectively for 20 min. The mixture was added to HeLa cells. Following 2 h incubation the cells were lysed and the CNF1-catalysed deamidation of RhoA was analyzed by the shift of the modified GTPase in SDS-PAGE by Western-blotting (A). HeLa cells were incubated with an anti Lu/BCAM antibody (AB B12) that binds to the extracellular domain or as control with an anti-Lu/BCAM antibody (AB C16) directed against the intracellular part of the glycoprotein. GST-CNF1 was then added to the cells for 2 h. We followed the toxins uptake by the amount of modified RhoA (shift in SDS-PAGE, B). Shown is a typical result of 3 independent experiments. Colocalization of DyLight488-labeled GST-CNF1 with Lu/BCAM and EEA1 (C) Top: HeLa-cells were treated on ice with DyLight488-labeled GST-CNF1 (5 mg/ml) (green) for 30 min to allow receptor binding. After 30 min cells were transferred to 37° for 30 min to induce uptake. Subsequently cells were fixed and stained for EEA1 (red) by immunofluorescence. Images are from the middle of the confocal stack. The image on the right is a maximal projection of all confocal planes. Scale bar indicates 10 µm. Middle: HeLa-cells were treated with labeled GST-CNF1 as in A. After fixation cells were stained for Lu/BCAM (red) by immunofluorescence. Images are from the middle of the confocal stack. The image on the right is a magnification of the white box. Scale bar indicates 10 µm. Bottom: HeLa-cells were treated as in A, but instead of GST-CNF1, cells were treated with DyLight488-labeled GST-CNFY (5 mg/ml) (green). After fixation cells were stained for Lu/BCAM (red) by immunofluorescence. Images are from the middle of the confocal stack. The image on the right is a magnification of the white box. Scale bar indicates 10 µm. Shown is a typical staining of 9 HeLa cells analyzed.

We asked whether Lu/BCAM is an alternative receptor in the absence of 67LR or whether binding to Lu/BCAM is generally crucial for toxin uptake. In the latter case, blocking the interaction of CNF1 with Lu/BCAM should inhibit the uptake of the toxin into cells. Uptake of CNF1 can be monitored by the shift of deamidated RhoA to higher molecular weight in SDS-PAGE [5] and by the morphological changes of cells induced by the toxin. To this end, we performed competition experiments with recombinant BCAM (rBCAM) and CNF1 on HeLa cells. We incubated the toxin with rBCAM (in a molar ratio CNF1∶rBCAM of 1∶1, 1∶10 and 1∶100, respectively) for 20 min and then treated cells with the mixture. As shown in Fig. 2A, preincubation of CNF with rBCAM inhibited uptake of the toxin in a concentration-dependent manner, indicating direct interaction of the proteins. In a second approach, we blocked the binding of CNF1 to Lu/BCAM directly on the cell surface. For these experiments, HeLa cells were treated with an anti Lu/BCAM antibody (AB B12) that binds to the extracellular domain of Lu/BCAM. As control we used an anti-Lu/BCAM antibody (AB C16) directed against the intracellular part of the glycoprotein. Toxin uptake was determined by the amount of modified RhoA (shift in SDS-PAGE) after 2 h of incubation. Fig. 2B shows that pre-incubation of cells with the antibody against Lu/BCAM extracellular domain inhibited the uptake of CNF1, whereas the control antibody had no effect. As expected, the antibodies added to the cells without the toxin had no effect. The data indicate that binding of CNF1 to Lu/BCAM is required for the uptake of the toxin into cells.

### Colocalization of Lu/BCAM with CNF1 but not with CNFY

To visualize CNF1 on the cell surface, we fluorescently labeled GST-CNF1 (green) and studied colocalization with Lu/BCAM using an antibody against the receptor and a second rhodamine labeled antibody (red). As negative control we used labeled GST-CNFY. Binding of the toxins was performed at 4°C and the cells incubated for further 30 min at 37°C to allow endocytosis. The cells were fixed and stained for Lu/BCAM. As shown in Fig. 2C CNF1 colocalized with Lu/BCAM, whereas there was no colocalization of CNFY with this receptor. Colocalization occurred in vesicular structures. It is known that CNF1 is released from endosomes following acidification [17]. Therefore, we confirmed the colocalization of CNF1 with the endosomal marker EEA1, which exclusively localizes to early endosomes. For immunofluorescence we used an antibody against EEA1 and a second rhodamine labeled antibody (red). As shown in Fig. 2C (top) the toxin was localized to early endosomes.

### Lu/BCAM is crucial for binding and uptake of CNF1

**Figure 3:**
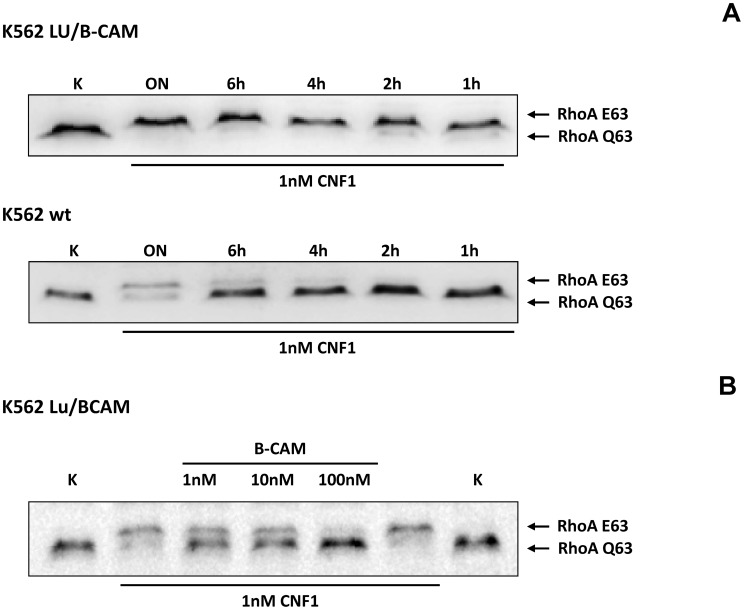
Cells not expressing Lu/BCAM do not respond to CNF1. A) K562 leukemia cells, which do not express Lu/BCAM (top) and the isogenic cell line K562-Lu/BCAM expressing the receptor (bottom), were treated with GST-CNF1 for different time periods from 1 h to overnight (ON) as indicated. Uptake of the toxin was analyzed by the shift of modified RhoA in SDS-PAGE. B) GST-CNF1 was incubated with buffer or with recombinant BCAM in a molar ratio CNF1∶rBCAM of 1∶1, 1∶10 and 1∶100, respectively for 20 min. The mixture was added to K562-Lu/BCAM cells for 2 h. Cells were lysed and the deamidation of RhoA was analyzed by the shift of the modified GTPase in SDS-PAGE by Western-blotting. Data are representative for at least 3 independent experiments.

To corroborate the requirement of Lu/BCAM for toxin binding and/or uptake we made use of human immortalized myelogenous leukemia cells (K562), which do not express Lu/BCAM. As control the same cell line stably expressing recombinant Lu/BCAM (K562-Lu/BCAM) was employed [18]. We verified expression of Lu/BCAM and analyzed the presence of 37LRP in the two cell-lines by Western-blotting. Whereas 37LRP is expressed in both cell lines, Lu/BCAM could only be detected in K562-Lu/BCAM but not in K562 cells (Fig. S2). To study the uptake of CNF1, leukemia cells and Lu/BCAM-expressing cells were treated with the toxin for different time periods and, subsequently, the uptake of CNF was analyzed by the shift of modified RhoA. The CNF1-induced shift of RhoA was incomplete in K562 cells not expressing Lu/BCAM even after overnight intoxication (Fig. 3A, bottom). By contrast, in Lu/BCAM expressing cells deamidation of RhoA occurred within 1 h (Fig. 3A, top). Apparently a low amount of toxin was taken up into the leukemia cells which do not express Lu/BCAM. This may occur by unspecific pinocytosis or by binding to the 67LR present on both cell lines. The data indicate that Lu/BCAM is essential for efficient uptake of CNF1. To further support this finding we performed competition experiments with rBCAM and CNF1 on K562-Lu/BCAM cells. We pre-incubated the toxin with rBCAM (in a molar ratio CNF1∶rBCAM of 1∶1, 1∶10 and 1∶100, respectively) for 20 min and treated the cells with the mixture. As expected, preincubation of CNF with rBCAM inhibited uptake of the toxin in a concentration-dependent manner (Fig. 3B).

We intended to distinguish between binding and uptake of the toxin. Therefore, we performed fluorescence activated cell sorting (FACS) analysis with DyLight488-labeled GST-CNF1. First, we added increasing amounts of labeled toxin to HeLa cells, incubated the cells for 15 min on ice and washed them with PBS. Afterwards we analyzed the fluorescence of the cells by FACS measurements. As expected, increasing concentrations of the labeled toxin shifted the cells to higher fluorescence, indicating specific binding of GST-CNF to the cells (Fig. S3, left). Data from 3 independent experiments were quantified (Fig. S3, right).

**Figure 4:**
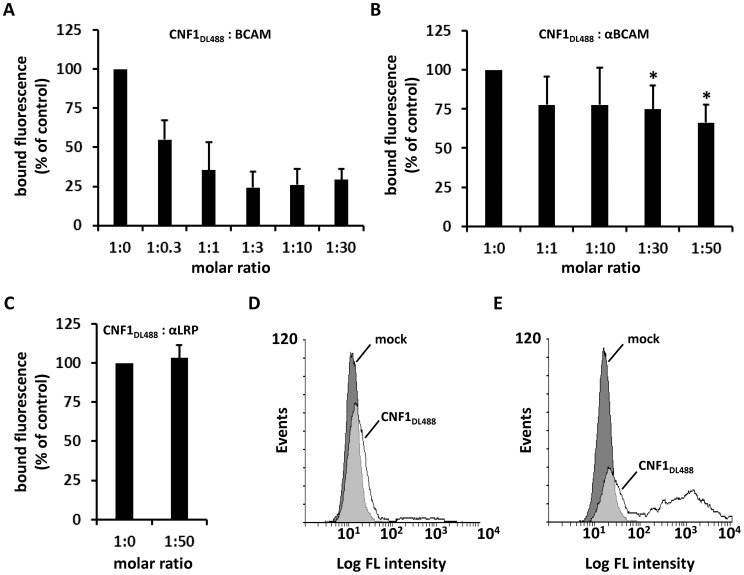
CNF1 directly interacts with Lu/BCAM on the cell surface. Suspensions of HeLa cells ((A–C) 1×10^5^ cells in 1 ml medium) were incubated for 20 min at 4°C with 2 µg DyLight488-labeled GST-CNF1 (CNF1DL488) alone or together with increasing amounts of rBCAM protein (A). Cells were then washed in PBS and subjected to FACS analysis. The experiment was repeated with 2 µg DyLight488-labeled GST-CNF1 (CNF1DL488) alone or together with increasing amounts of αLu/BCAM antibody (molar ratio CNF∶antibody up to 1∶50, * p<0.05) (B) or a 50-fold excess of the αLRP antibody as indicated (C). Data are presented as % fluorescence of control with CNF1DL488 set to 100%. Non- (D) and Lu/BCAM-expressing (E) K562 cells were either left untreated (mock) or were incubated with 2 µg CNF1DL488 for 20 min at 4°C and, following washing with PBS, subjected to FACS analysis. Results are presented as histogram plots, where single cell events are plotted against cell surface-bound fluorescence (Log FL intensity).

We further studied the toxin Lu/BCAM interaction and performed competition experiments firstly with recombinant BCAM and in a second approach with antibodies. We either used increasing amounts of an anti-Lu/BCAM antibody directed against the extracellular domain of the receptor or an antibody directed against 37LRP. All competitors were used in increasing amounts (molar ratio CNF1∶competitor from 1∶0 to 1∶50, respectively as indicated). The fluorescence decreased to lower levels in the presence of the competitors rBCAM (Fig. 4A) and anti-Lu/BCAM (Fig. 4B), respectively but not in the presence of a 50-fold molar excess of anti-37LRP (Fig. 4C). Whereas GST-CNF1 binding was decreased with recombinant BCAM to about 25% of control, the effect was less pronounced but significant with the antibody directed against Lu/BCAM. No change in fluorescence was detected in the presence of the antibody against 37LRP. The data indicate that the toxin specifically binds to Lu/BCAM.

To verify binding of the toxin to Lu/BCAM, we performed FACS analysis with K562 cells (Fig. 4D) and with receptor expressing K562-Lu/BCAM cells (Fig. 4E). At first cells were incubated with DyLight488-labeled GST-CNF1 (2 µg/mL), washed and analyzed. In line with our precipitation experiments, K562 cells only marginally shifted to higher fluorescence, whereas the K562-Lu/BCAM cells showed a broad spectrum of fluorescent cells ranging from low to high fluorescence. The latter may correspond to the degree of Lu/BCAM overexpression in these cells, which seems to vary from cell to cell. Pre-incubation of labeled GST-CNF1 with rBCAM concordantly reduced the amount of toxin bound to these cells but did not completely block CNF-binding. This effect may be based on the high amount of Lu/BCAM expressed.

### Role of Lu/BCAM glycosylation

Lu/BCAM is known to be modified by glycosylation. Four N-glycosylation sites have been identified, which account for approximately 15% weight composition of the protein [13]. Some bacterial toxins are recruited to cellular membranes by binding to sugar chains before a high affinity interaction with a surface protein occurs. This has been shown for example for tetanus neurotoxin [19]. In our co-precipitation assay (Fig. 1) only the high molecular weight form of Lu/BCAM was enriched by binding to CNF1, whereas the lower molecular weight form was not precipitated with CNF, although it appeared as the most prominent band in the lysate. This indicates that N-glycosylation may be important for interaction with the toxin. Therefore, we tested, whether N-glycosylation of Lu/BCAM plays a role for CNF1 binding. The recombinant BCAM used in this study was purified from Lu/BCAM-overexpressing mammalian cells. The protein is fully glycosylated. We treated recombinant BCAM with PNGaseF and analyzed deglycosylation by SDS-PAGE. Deglycosylated rBCAM should run lower according to its lower molecular weight. As shown in Fig. S4A, PNGaseF-treated rBCAM runs at a lower molecular weight (67 kDa) as compared with the glycosylated rBCAM (84 kDa). This is indicative of effective deglycosylation [14]. We spotted CNF1 on nitrocellulose and repeated the overlay assay as described above with glycosylated recombinant BCAM and PNGaseF-treated deglycosylated rBCAM. In this assay CNF1 still interacts with deglycosylated rBCAM (Fig. S4B). Moreover, treatment of cells with PNGaseF and subsequent FACS-analysis revealed that the toxin binds with even higher affinity to the cells (Fig. S4C). This may be due to a higher accessibility of the protein part of the receptor. The data indicate that N-glycosylation is not necessary for the interaction of CNF1 with Lu/BCAM. The exclusive precipitation of the high molecular weight form of the protein from cells may indicate that this is the surface exposed form of Lu/BCAM.

### Defining the CNF1-Lu/BCAM interaction site

Two sites of CNF1 have been described to be necessary for its interaction with mammalian cells: The N-terminal receptor binding domain (aa 1–342, [16]) and a small part of CNF1 adjacent to the catalytic domain (aa 683–730, [16]).

**Figure 5:**
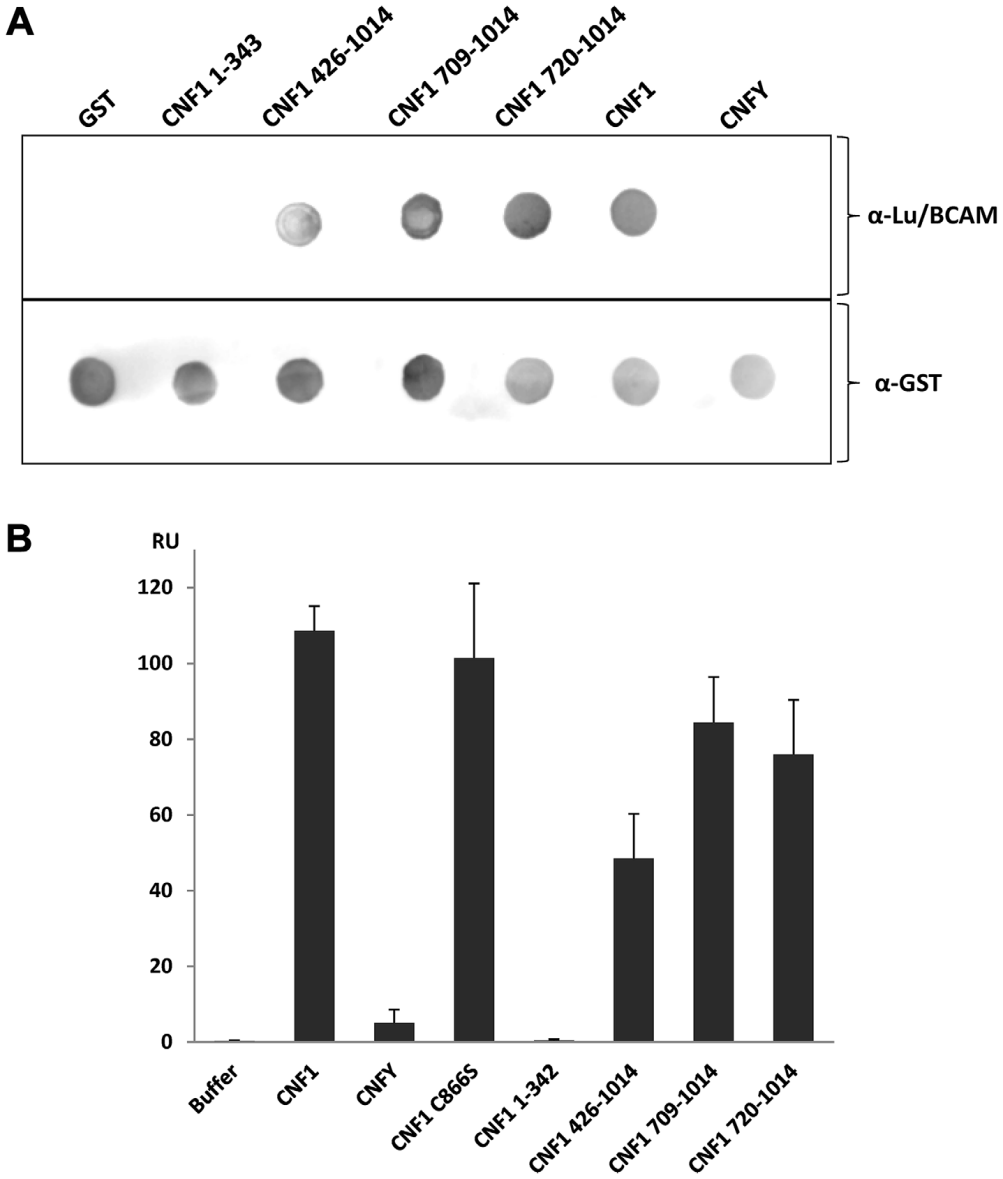
Direct rBCAM-CNF interaction. A) For dot-blots 5 µl of 3 µM solutions of GST-CNFs, GST-CNF fragments and GST alone, respectively, were spotted onto a nitrocellulose membrane. The membrane was blocked with skimmed milk and recombinant BCAM (6 µM) was added for 1 h at room temperature. Following washing bound rBCAM was detected with an anti-Lu/BCAM antibody. Equal protein load was analyzed by visualizing the GST part of the spotted proteins with an anti GST-antibody. B) Biacore protein-protein interaction studies: An antibody against human IgG (Millipore) was coupled to two lanes of a CM5-biacore chip. As ligand recombinant BCAM containing a C-terminal human IgG domain (Sino biologics) was exclusively guided over lane 2. In a second step, GST-CNF proteins as analyte were guided over both lanes. Bound protein is given as relative units (RU) corrected for the unspecific binding to lane 1 as average plus standard deviation of three independent experiments.

To narrow down the region of CNF1, which interacts with Lu/BCAM, at first we studied direct protein-protein interaction. We performed dot-blot and surface plasmon resonance (Biacore) studies with recombinant BCAM and several CNF1 fragments. Additionally, we used the CNF-family member CNFY, which is identical in length and shares amino acid identity to CNF1 of 61% (CNFY) [20]. All proteins were tested for correct folding/activity in an *in vitro* Rho shift assay, which indicates deamidation of recombinant RhoA. As expected, only the N-terminal fragment of CNF1 (aa 1–342), which does not contain the catalytic domain, did not catalyze the deamidation of RhoA (Fig. S5). For dot-blots, we spotted 3 µM solutions of GST-CNFs, GST-CNF fragments and GST alone, respectively, onto nitrocellulose. After membrane blockage, recombinant BCAM was added and bound rBCAM was detected using anti-BCAM antibody. Equal protein load was analyzed by visualizing the GST part of the spotted proteins with an anti GST-antibody. As shown in Fig. 5A-top, significantly more rBCAM bound to CNF1 as compared with CNFY. In contrast, rBCAM did not interact with GST. The data indicate that CNF1 binds with higher affinity to rBCAM as compared to CNFY. This is in line with previous competition experiments, which indicate that CNF1 and CNFY apparently bind to a different receptor [17], [20]. Moreover, the binding site seems to be located in close proximity to the catalytic domain. The CNF1 fragments 426–1014, 709–1014 and 720 to 1014 exhibited strong binding to rBCAM, indicating that amino acids 720 to 1014 of CNF1 are sufficient for the interaction with this receptor (5A-bottom). In contrast, the N-terminal part (aa 1–342), which was previously suggested to be responsible for CNF-receptor interaction, did not interact with rBCAM.

**Figure 6:**
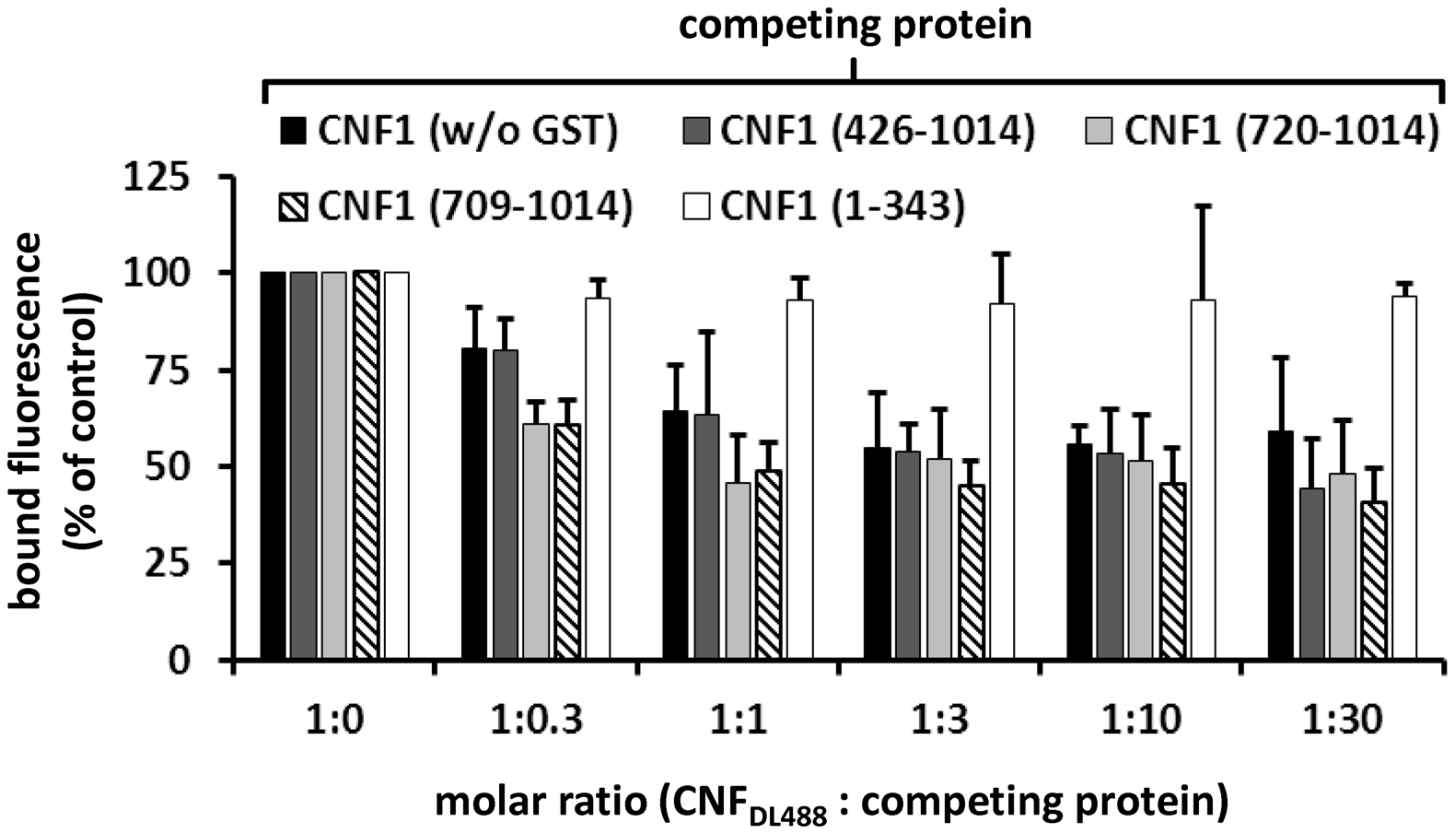
CNF1 binds to the cell surface via a C-terminal peptide. Suspensions of HeLa cells (1×10^5^ cells in 1 ml medium) were incubated for 20 min at 4°C with 2 µg DyLight488-labeled GST-CNF1 (CNF1DL488) alone, or together with increasing amounts (up to 30 fold) of untagged non-labeled CNF1 and the non-labeled fragments GST-CNF1-(426–1014), GST-CNF1-(720–1014), GST-CNF1-(709–1014) and GST-CNF1-(1–343), respectively. Following washing with PBS, cells were subjected to FACS analysis. Results are presented as mean of cell surface-bound fluorescence (% of control) of three independent experiments+standard deviation.

For quantitative analysis of the protein-protein interaction we used plasmon resonance spectroscopy (Biacore). For these studies we used recombinant Lu/BCAM, which contains a C-terminal human IgG domain for purification (Sino biology). We covalently coupled an antibody against human IgG to two lanes of a CM5-biacore chip. For analysis of the rBCAM-CNF1 interaction, rBCAM as ligand (only to lane 2) and in a second step, CNF proteins as analyte (both lanes) were guided over the lanes. This allows for correction of unspecific protein binding to the antibody. In Fig. 5B bound protein is given as relative units (RU) corrected for the unspecific binding to lane 1. The data show that the strongest binding to rBCAM occurs with the full-length toxin and with the C-terminal part of CNF1, whereas no interaction with rBCAM could be detected with CNFY or with the N-terminal part of CNF1 (aa 1–342). The fragment 426–1014 was weaker in its interaction to Lu/BCAM as compared to the C-terminal fragments 709–1014 or 720–1014. The difference between CNF1 (aa 426–1014) and the shorter fragment may be based on different protein stability or better accessibility of the receptor binding domain in the shorter fragments which needs further investigation. The data suggest that the interaction site of CNF1 with Lu/BCAM is located within amino acids 720 to 1014 and does not occur with the N-terminus (aa 1–342). To confirm these results, we repeated the FACS experiments as described above with labeled GST-CNF1 and increasing amounts of unlabeled CNF1 fragments (molar ratio CNF1∶competitor from 1∶0 to 1∶30, respectively as indicated). Moreover, we used untagged CNF1 for competition. As expected, the C-terminal fragments of the toxin (aa 709–1014 and aa 720–1014) were able to inhibit binding of CNF1 to the cells whereas pre-incubation with the N-terminus (aa 1–342) had no effect (Fig. 6). In line with the dot-blot analysis, the fragment 426–1014 was weaker in its interaction to Lu/BCAM as compared to the C-terminal fragments.

### CNF1 without the N-terminus is not sufficient for the intoxication of cells

**Figure 7:**
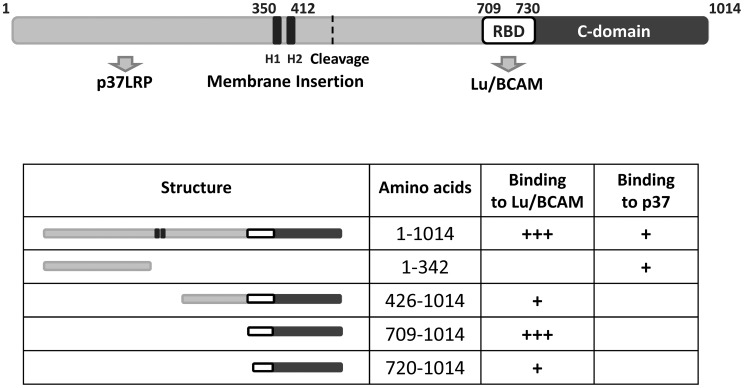
Summary of the CNF1 domain structure. CNF1 consists of 1014 amino acids. We found amino acids 1–342 responsible for interaction with the laminin receptor precursor (37LRP). Binding to Lu/BCAM occurs with amino acids 720 to 1014. Data from the literature suggest amino acids 683–730 to mediate binding to mammalian cells [16] indicating this part to be responsible for BCAM binding (white box, RBD). The C-domain represents the catalytic part of the toxin. The two hydrophobic helices (H1 and H2) are crucial for insertion into the endosomal membrane.

The catalytic part of CNF1 has been localized to amino acids 720 to 1014 [21]. Our data show that the binding site of the toxin to Lu/BCAM is located adjacent to this part and even overlap. Therefore, we tested, whether the toxin deleted for the N-terminus but containing a translocation domain, receptor binding and catalytic part may be sufficient for intoxication of cells. We incubated HeLa cells with the CNF1 fragment (aa 343 to 1014), which contains the catalytic domain, the Lu/BCAM binding site and additionally the two hydrophobic regions H1 and H2 suggested to mediate insertion into the endosomal membrane [22]. We lysed the cells and analyzed the modification of RhoA using the Rho shift assay. Even a high amount of the toxin part (1,5 µM) added to mammalian cells was not sufficient to intoxicate the cells (not shown). This indicates that besides the CNF1-Lu/BCAM interaction, binding to p37LRP is required for the intoxication of the cells. One explanation could be that the protein is not released into the cytosol. However, further experiments are required, to analyze this feature. Our data are summarized in Fig. 7.

## Discussion

Our study sheds new light on the uptake mechanism of the bacterial toxin CNF1. It is widely accepted that the toxin enters the cytosol of mammalian cells from endosomes, following receptor-mediated endocytosis [23]. Inhibition of endosome acidification with bafilomycin A completely blocks intoxication of cultured cells [17], [24]. Here, we identified Lu/BCAM as a crucial receptor for the toxin. For precipitation of CNF1-interacting proteins, we used a method recently employed for the identification of the alpha-toxin receptor [25]. Following binding to the surface of living HeLa cells and subsequent lysis, we isolated the toxin-protein complexes, using magnetic beads. This method ensures correct folding and orientation of membrane proteins during toxin binding. Moreover, intracellular proteins and cytosolic domains of membrane proteins are excluded as interaction partners. MALDI-TOF analysis of the precipitate identified Lu/BCAM as interaction partner for CNF1. We verified CNF1 Lu/BCAM interaction by several methods:

Western-blotting revealed that Lu/BCAM was exclusively co-precipitated with CNF1 but not with CNFY. Second, dot-blot analysis with several CNF1 fragments and recombinant BCAM delineated the site of interaction to the C-terminus (aa 720 to 1014). Finally, we supported our findings with surface plasmon resonance measurements (Biacore). In this system we were not able to displace CNF1 from BCAM using several conditions like high salt buffer (500 mM NaCl, 20 mM Tris/HCl, pH 7.4) basic or acidic buffer (50 mM NaOH, 10 mM glycine, pH 2.5). Therefore, it was not possible to calculate the dissociation constant (Kd) of the rBCAM-CNF interaction.

Binding to Lu/BCAM occurred with amino acids 720–1014 with comparable affinity as determined for the full-length protein. Using FACS analysis, we show that this part of the toxin is sufficient for interaction with the cell surface. Moreover, it is sensitive for competition with antibodies against Lu/BCAM or with an excess of recombinant BCAM. In previous studies the non-integrin laminin receptor precursor 37LRP has been identified as interaction partner for the N-terminal part of CNF1 in a yeast two-hybrid screen [8]. This part has been suggested as the toxins receptor binding domain, because an excess of a corresponding CNF1 peptide incubated together with full-length CNF1 blocks intoxication of cells [21]. In line with this, the N-terminal binding partner 37LRP/67LR seems to be required for the action of the toxin. It has been shown that 37LRP/67LR is important for intoxication of mammalian cells and for the opening of the brain barrier in a mouse model [26], [27]. No direct binding to the cell surface was proven and p37LRP/p67 may have other functions like transport into the cell.

Surprisingly, we could not find any interaction with Lu/BCAM and the previously suggested N-terminal receptor binding site. This suggests that 37LRP/67LR and Lu/BCAM are not alternative receptors, although they are both laminin-binding proteins. We found Lu/BCAM to be crucial for binding, because cells deficient in Lu/BCAM but expressing LRP could not bind labeled CNF1. This indicates that 37LRP/67LR is not sufficient for toxin binding. Therefore, we conclude that 37LRP/67LR and Lu/BCAM are both required for toxin action but with different functions. We suggest two interaction sites in CNF1: The N-terminus binding to 37LRP/67LR as postulated by Kim [8], [26] and the C-terminus (probably a region around amino acids 683–730, [16]) binding with high affinity to Lu/BCAM. In line with this, two different cell interaction sites of CNF1 have been identified by McNichol and coworkers, which both are necessary for efficient intoxication of cells [16]. Our data show that Lu/BCAM is required for toxin binding. However, it is not sufficient for intoxication of mammalian cells, since CNF1 deleted for the N-terminal 342 amino acids can bind to cells but is not able to intoxicate them. What is the role of the N-terminus and its interaction with 37LRP/67LR? We observed that the N-terminal part of CNF1 forms SDS stable tetramers, although only a small fraction of the purified protein was found in the oligomer-band (data not shown). Further studies are required to analyze a possible oligomerization of CNF1. We speculate that 37LRP/67LR may bind to the pre-formed oligomer and may play a crucial role for the uptake of CNF1 into the cytosol. Initial binding of the toxin to the cell surface requires Lu/BCAM. It remains to be analyzed, whether interaction of an oligomer is required for pore-formation in the endosomal membrane and/or for transport of the catalytic domain into the cytosol.

Taken together, we present a novel model of the action of CNF1, which is outlined in Fig. 7. We suggest that CNF1 binds to the surface of mammalian cells with its receptor binding domain located adjacent to the catalytic domain. The crystal structure of the catalytic domain (aa 720–1014) reveals an additional helix (aa 720 to 734) sticking out of the otherwise globular structure (735 to 1014). This part does not directly contribute to the catalytic core domain of the toxin [28] but may connect the receptor binding domain of CNF1 with the catalytic domain or even is part of the Lu/BCAM interaction site. Further studies are required to analyze this important feature.

We have shown recently that cleavage of CNF1 enhances its biologic activity and that this cleavage does not occur in the cytosol but either on the cell surface or within endosomes [29]. Two surface binding sites (one at the N-terminus and the other near the C-terminus) would allow toxin cleavage on the membrane/vesicle surface without losing the interaction of separated toxin parts. This allows subsequent pore formation and translocation. Thus, while some toxins (e.g., diphtheria toxin) are functionally connected by SS-bridges, CNF might use two cell surface binding sites for efficient up-take.

## Methods

### Cell culture

HeLa and Hek293 cells were grown in Dulbecco's modified Eagle's medium (DMEM) supplemented with 10% fetal calf serum, 1% nonessential amino acids, penicillin (4 mM), and streptomycin (4 mM).

K562 wt and K562 Lu/BCAM (provided by Anne Filipe, INSERM Paris) were grown in RPMI (RPMI-1640) containing 10% fetal calf serum, penicillin (4 mM) and streptomycin (4 mM). K562 cells expressing Lu/BCAM were grown in medium containing 1 mM Geniticin. T47D cells were cultured in DMEM supplemented with 10% fetal calf serum, 1% nonessential amino acids, penicillin (4 mM), and streptomycin (4 mM) and 80 iU/l insulin.

All cell lines were cultivated in a humidified atmosphere containing 5% CO_2_ at 37°C.

For competition experiments cells were grown to subconfluency and treated with appropriate amounts of toxin for 1 to 16 h, as indicated. For competition assays cells were pre-incubated with antibodies against Lu/BCAM, or the toxins were pre-treated with recombinant BCAM (Sinobiologics) prior to intoxication as indicated.

Cell lysates were generated as follows: Cells were washed twice with phosphate buffered saline (PBS) and lysed with GST-Fish buffer (10% glycerol, 50 mM Tris/HCl, pH 7.4, 100 mM NaCl, 1% NP-40, 2 mM MgCl_2_ and 1 mM PMSF). Lysates were cleared by centrifugation (20 min, 21,000 g, 4°C). To separate cytosolic and membrane fractions, lysates were centrifuged (1 h, 100,000 g, 4°C). Membrane pellets were dissolved in hot sample buffer (100 mM Tris (pH 6.8), 10% sodium dodecyl sulfate (SDS), 10% glycerol, 100 mM dithiotreitol (DTT)).

### Sample preparation, LC-MS/MS and data analysis

In-gel digests were performed as described in standard protocols. Briefly, the excised gel bands were destained with 30% ACN, shrunk with 100% ACN, and dried in a Vacuum Concentrator (Concentrator 5301, Eppendorf, Hamburg, Germany). Digests with trypsin were performed overnight at 37°C in 0.1 M NH_4_HCO_3_ (pH 8). About 0.1 µg of protease was used for one gel band. Peptides were extracted from the gel slices with 5% formic acid.

All LC-MS/MS analyses were performed on an ion trap mass spectrometer (Agilent 6340, Agilent Technologies) coupled to an 1200 Agilent nanoflow system via a HPLC-Chip cube ESI interface. Peptides were separated on a HPLC-Chip with an analytical column of 75 µm i.d. and 150 mm length and a 40-nL trap column both packed with Zorbax 300SB C-18 (5 µm particle size). Peptides were eluted with a linear acetonitrile gradient with 1%/min at a flow rate of 300 nL/min (starting with 3% acetonitrile).

MS/MS analyses were performed using data-dependent acquisition mode. After a MS scan (standard enhanced mode), a maximum of three peptides were selected for MS/MS (CID, standard enhanced mode). The automated gain control was set to 350000. The maximum accumulation time was set to 300 ms.

Mascot Distiller 2.1 (Matrix Science, UK) was used for raw data processing and for generating peak lists, essentially with standard settings for the Agilent ion trap. Mascot Server 2.2 (Matrix Science, UK) was used for database searching with the following parameters: peptide mass tolerance: 1.1 Da, MS/MS mass tolerance: 0.3 Da, 13C: 1, enzyme: trypsin with max. 2 missed cleavage, variable modifications: Gln→pyroGlu (N-term. Q), oxidation (M) and carbamidomethyl (C). The SwissProt database was used for database searching. Scaffold 4.0.4 (Proteome Software, USA) was used for statistical analysis and filtering of the search results (protein threshold: 99%, peptide threshold: 80%, minimum number of peptides: 2).

### Purification of 37LRP/67LR

The 67LR was purified from T47D human breast carcinoma cells (obtained from Marc Hirschfeld, Albert-Ludwigs-Universität; Frauenklinik).

After rotational incubation these cells shed the receptor into the medium.

Cells were grown up to 70–75% confluency, washed with calcium-free PBS and harvested by trypsination for 5 minutes with TE (Trypsin-EDTA).

The cells were centrifuged at 800 *g* for 10 minutes. Approximately 2×10^7^ cells were suspended in 1 ml ice-cold PBS and incubated for 4 h at 37°C under rotation. After removal of the cells by centrifugation at 16000 *g* for 15 min the shed receptor was precipitated from the supernatant with acetone or frozen at −80°C.

### SDS-PAGE and western-blotting

Cell lysates were separated by 12.5% SDS-PAGE (for RhoA-shift assay 15% urea-SDS-PAGE) and transferred onto a polyvinylidene difluoride membrane. Membranes were either blocked with skimmed milk (5%) or bovine serum albumin (BSA, 3%) for 1 h at room temperature. Proteins were detected using anti-CNF1 monoclonal antibody (1∶3000; Santa Cruz), anti-RhoA monoclonal antibody (1∶500; Santa Cruz), anti-Lu/BCAM (1∶2500; Abcam; 1∶1000; Santa Cruz) or anti-LRP (1∶1000, Santa Cruz) and a respective horseradish peroxidase-coupled second antibody. Detection occurred by enhanced chemiluminescence.

### Immunoprecipitation

Hek293 and HeLa cells (2–3 subconfluent 15 cm dishes) were incubated with GST-CNF1-GST, GST-CNFY-GST or GST (500 ng/ml, respectively) for 20 min at 4°C. Cells were harvested and lysed and the IP was conducted using anti-GST magnetic beads according to the manufacturers manual (Miltenyi Biotech).

### Dot-blot

CNF proteins in given concentrations were spotted onto a nitrocellulose membrane at 4°C. After drying, membranes were incubated with 5 nM recombinant BCAM in TBST, 30 min at 4°C and blocked in 5% milk for 1 h at room temperature. The membranes were washed 3 times with TBST (Tris buffered saline; 0.05% Tween-20). Binding of rBCAM was detected using anti-Lu/BCAM antibodies (1∶2500; Abcam). GST served as control and was detected with anti-GST antibodies (1∶500; Santa Cruz). Detection occurred by enhanced chemiluminescence with horseradish peroxidase-coupled second antibodies.

### Surface plasmon resonance (Biacore)

An antibody against human IgG (Santa Cruz, 20 µg/mL) was covalently coupled to two lanes of a CM5-biacore chip with 400 mM N-ethyl-N-dimethylaminopropyl-carbodiimide (EDC) and 100 mM N-hydroxy-succinimide (NHS). The lanes were saturated with 1M ethanolamine. As ligand recombinant BCAM containing a C-terminal human IgG domain (0.2 µM) was exclusively guided over lane 2. In a second step, CNF proteins as analyte (1 µM) were guided over both lanes. Bound protein was determined as relative units (RU) corrected for the unspecific binding to lane 1. Regeneration occurred with 10 mM glycine/HCl, pH 2.5.

### FACS

Cells were detached from culture dishes by incubation with 10 mM EDTA/PBS. The cell suspension was washed twice with PBS and kept on ice. 250,000 cells were incubated 20 min at 4°C with different concentrations of labeled toxins, and fluorescence was measured with a fluorescence-activated cell sorter (FACS) using the BD FACSCalibur platform. Cell surface-bound fluorescence was detected with an argon-ion laser (488 nm) and the 530-nm-band-pass filter (FITC). Toxins were labeled according to the manufacturer's manual (DyLight 488 carboxylic acid succinimidyl ester (Invitrogen).

For competition assays cells were either pre-incubated with anti-Lu/BCAM (Santa Cruz) or the toxins were pre-incubated with recombinant BCAM (Sinobiologics).

### Immunostaining

Cells were washed with phosphate buffered saline (PBS) and prefixed with ice cold methanol supplemented with 1 mM EGTA. After 10 min cells were transferred to 4% formaldehyde in PBS, washed, permeabilized with 0.15% Triton X-100 in PBS for 10 min and blocked by 1% BSA in PBS for 30 min. Incubation with the primary antibody (anti-EEA1 1∶200, Santa Cruz) (anti-Lu/BCAM 1∶150, Santa Cruz) was overnight at 4°C in PBS. Cells were washed with PBS and incubated with the suitable secondary antibody in PBS for 1 h at room temperature. Cells were washed, dried and embedded with Mowiol supplemented with DABCO (Sigma, St. Louis, MO, USA). Cells were analyzed with an inverted Axiovert 200 M microscope (Carl Zeiss GmbH, Jena, Germany), driven by Metamorph imaging software (Universal Imaging, Downingtown, PA, USA), with a Yokogawa CSU-X1 spinning disc confocal head (Tokyo, Japan) with emission filter wheel, with a Coolsnap HQ II digital camera and with 488 nm and 561 nm laser lines. Images were processed with Metamorph software.

## Supporting Information

Figure S1 Maldi-TOF analysis of co-precipitated proteins. Hek293 cells were incubated with GST-CNF1-GST, GST-CNFY-GST or GST for 20 min at 4°C. Cells were harvested and lysed and the IP was conducted using anti-GST magnetic beads according to the manufacturers manual (Miltenyi Biotech). Proteins were separated by SDS-PAGE and analyzed by MALDI-TOF analysis.
(TIFF)

Figure S2 Presence of 37LRP/67LR and Lu/BCAM in HEK293, HeLa, K562 and K562-LU/BCAM cells. Cell lysates were separated by SDS-PAGE and blotted onto a PVDF membrane. 37LRP/67LR and LU/BCAM were detected with specific antibodies.
(TIFF)

Figure S3 FACS-based analysis of CNF1 binding to HeLa cells. Suspensions of HeLa cells (1×10^5^ cells in 1 ml medium) were incubated for 20 min at 4°C with indicated concentrations of DyLight488-labeled GST-CNF1 (CNF1_DL488_), washed with PBS, and subjected to FACS analysis. Left: Results are presented as histogram plots, where single cell events are plotted against cell surface-bound fluorescence (Log FL intensity). Right: Data from 3 independent experiments (3.5 to 35 nM CNF1_DL488_) were quantified and are presented as arbitrary units (AU)+standard deviation.
(TIFF)

Figure S4 Role of Lu/BCAM glycosylation. Recombinant BCAM was treated with PNGaseF and analyzed deglycosylation by SDS-PAGE. De-glycosylated rBCAM runs faster according to its lower molecular weight (67 kDa) as compared with the glycosylated BCAM (84 kDa) (A). GST-CNF1, GST-CNFY and GST were spotted onto a nitrocellulose membrane. An overlay assay with glycosylated recombinant BCAM and PNGaseF-treated, de-glycosylated rBCAM was performed. Following washing bound rBCAM was detected with an anti-Lu/BCAM antibody. Equal protein load was analyzed by visualizing the GST part of the spotted proteins with an anti GST-antibody (B). Facs-analysis revealed that the toxin binds with higher affinity to the cells (C): Suspensions of PNGase F-treated (white, dashed lined peaks) or untreated (dark grey peaks) HEK293 cells (1×10^5^ cells in 1 ml medium) were incubated for 20 min at 4°C with 2 µg of DyLight488-labeled GST-CNF1 (CNF1_DL488_) or without protein (mock), washed with PBS, and subjected to FACS analysis. Results are presented as histogram plots, where single cell events are plotted against cell surface-bound fluorescence (Log FL intensity).
(TIFF)

Figure S5 Recombinant CNF1 fragments are folded correctly. Recombinant RhoA (5 µM) was incubated with GST-CNF1 and GST-CNF1 fragments (each 1 µM), respectively as indicated in a buffer, containing 50 mM TRIS-HCl, pH 7.5, 5 mM MgCl_2_, 1 mM EDTA, and 1 mM DTT for 4 h at 37°C. Proteins were loaded onto 12.5% SDS-gel containing 1 M urea. The samples were analyzed for the typical shift of deamidated RhoA to higher molecular weight.
(TIFF)
