# Correction: Lu/BCAM Adhesion Glycoprotein Is a Receptor for *Escherichia coli* Cytotoxic Necrotizing Factor 1 (CNF1)

**DOI:** 10.1371/annotation/6eec6403-e090-4283-aa34-34cc58ca0bbb

**Published:** 2014-01-28

**Authors:** Marianne Piteau, Panagiotis Papatheodorou, Carsten Schwan, Andreas Schlosser, Klaus Aktories, Gudula Schmidt

The following is a statement that is missing from the funding information in the published article:

"The article processing charge was funded by the open access publication fund of the Albert Ludwigs University Freiburg." 

