# Supplementary figures and images for: Lu/BCAM Adhesion Glycoprotein Is a Receptor for Escherichia coli Cytotoxic Necrotizing Factor 1 (CNF1)
Source: PLoS Pathog. 2014 Jan 16;10(1):e1003884. doi: 10.1371/journal.ppat.1003884 (PMC3894216; doi:10.1371/journal.ppat.1003884)

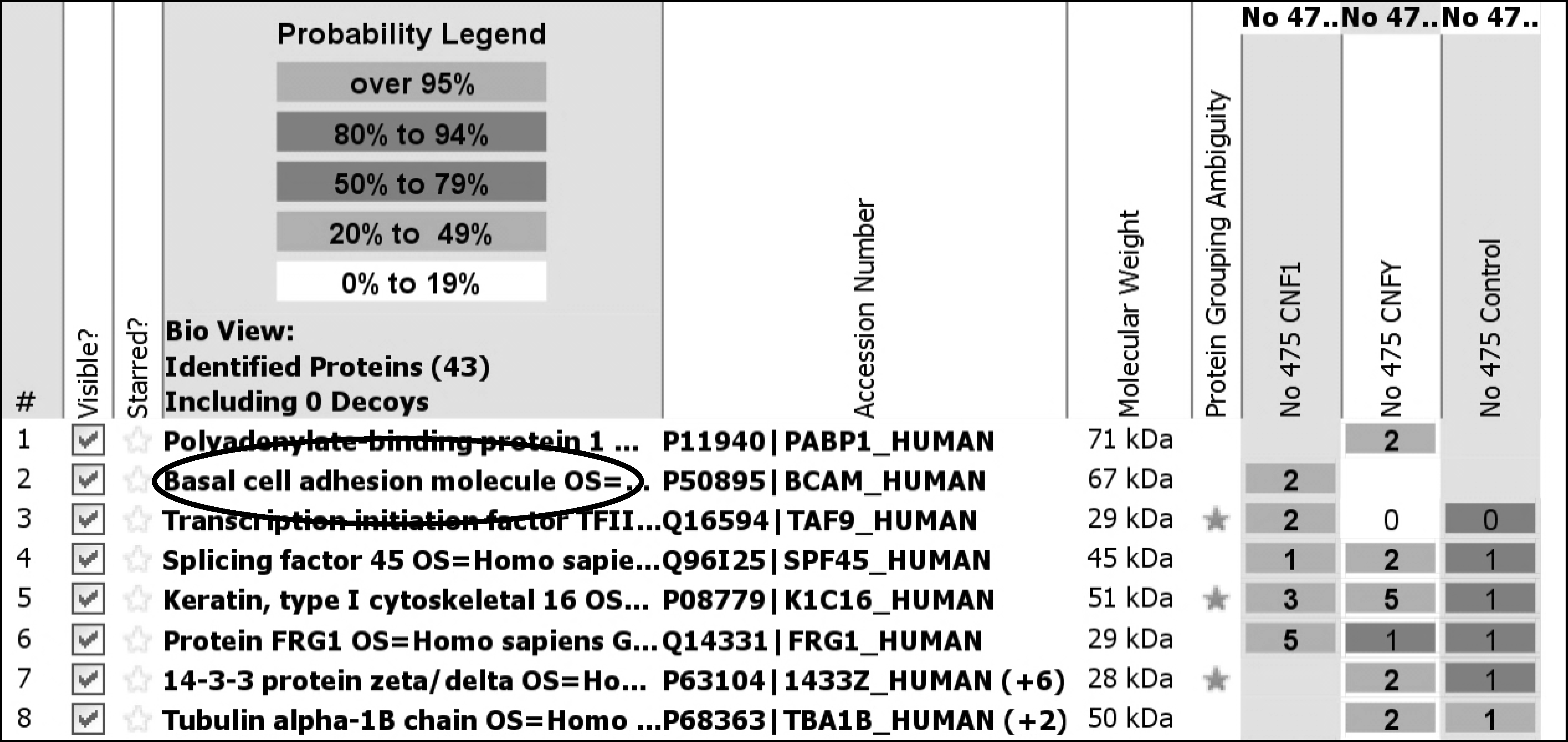

Supplement: Figure S1 — Maldi-TOF analysis of co-precipitated proteins. Hek293 cells were incubated with GST-CNF1-GST, GST-CNFY-GST or GST for 20 min at 4°C. Cells were harvested and lysed and the IP was conducted using anti-GST magnetic beads according to the manufacturers manual (Miltenyi Biotech). Proteins were separated by SDS-PAGE and analyzed by MALDI-TOF analysis. (TIFF) [file ppat.1003884.s001.tiff]

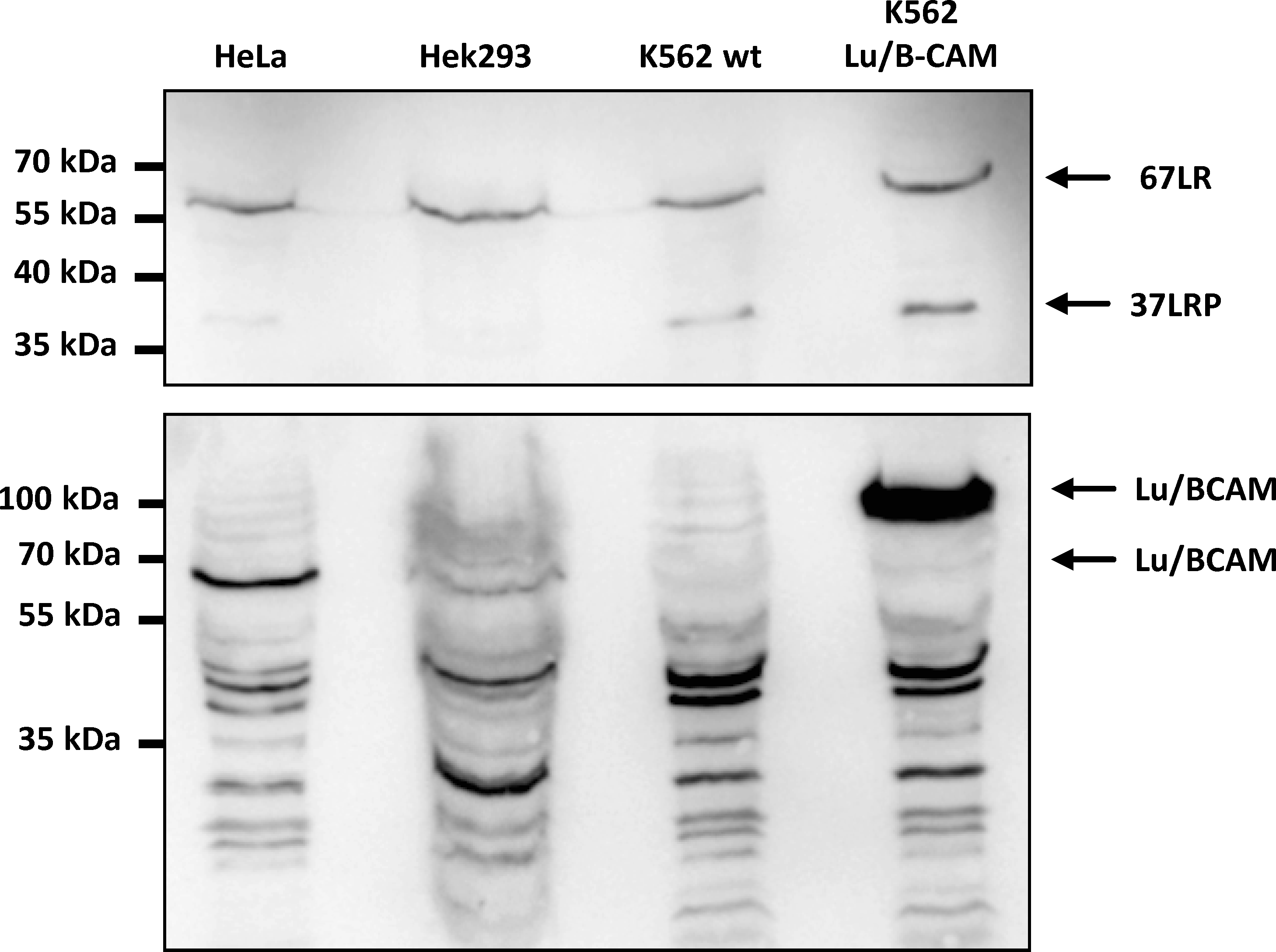

Supplement: Figure S2 — Presence of 37LRP/67LR and Lu/BCAM in HEK293, HeLa, K562 and K562-LU/BCAM cells. Cell lysates were separated by SDS-PAGE and blotted onto a PVDF membrane. 37LRP/67LR and LU/BCAM were detected with specific antibodies. (TIFF) [file ppat.1003884.s002.tiff]

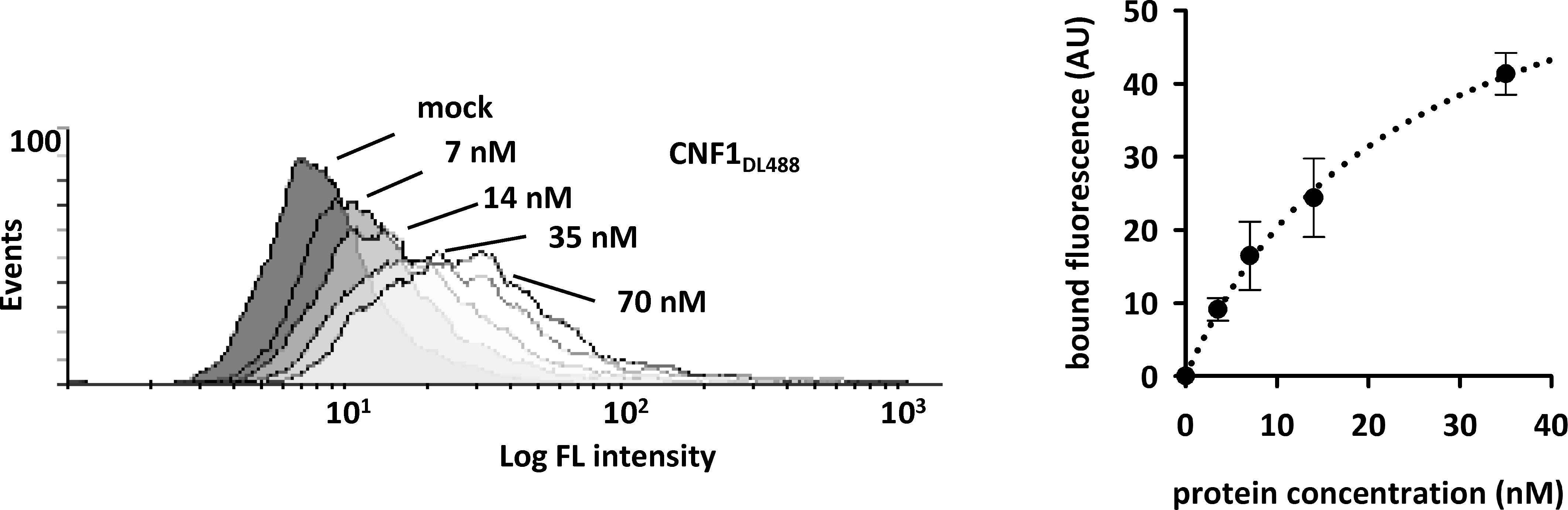

Supplement: Figure S3 — FACS-based analysis of CNF1 binding to HeLa cells. Suspensions of HeLa cells (1×10^5^ cells in 1 ml medium) were incubated for 20 min at 4°C with indicated concentrations of DyLight488-labeled GST-CNF1 (CNF1DL488), washed with PBS, and subjected to FACS analysis. Left: Results are presented as histogram plots, where single cell events are plotted against cell surface-bound fluorescence (Log FL intensity). Right: Data from 3 independent experiments (3.5 to 35 nM CNF1DL488) were quantified and are presented as arbitrary units (AU)+standard deviation. (TIFF) [file ppat.1003884.s003.tiff]

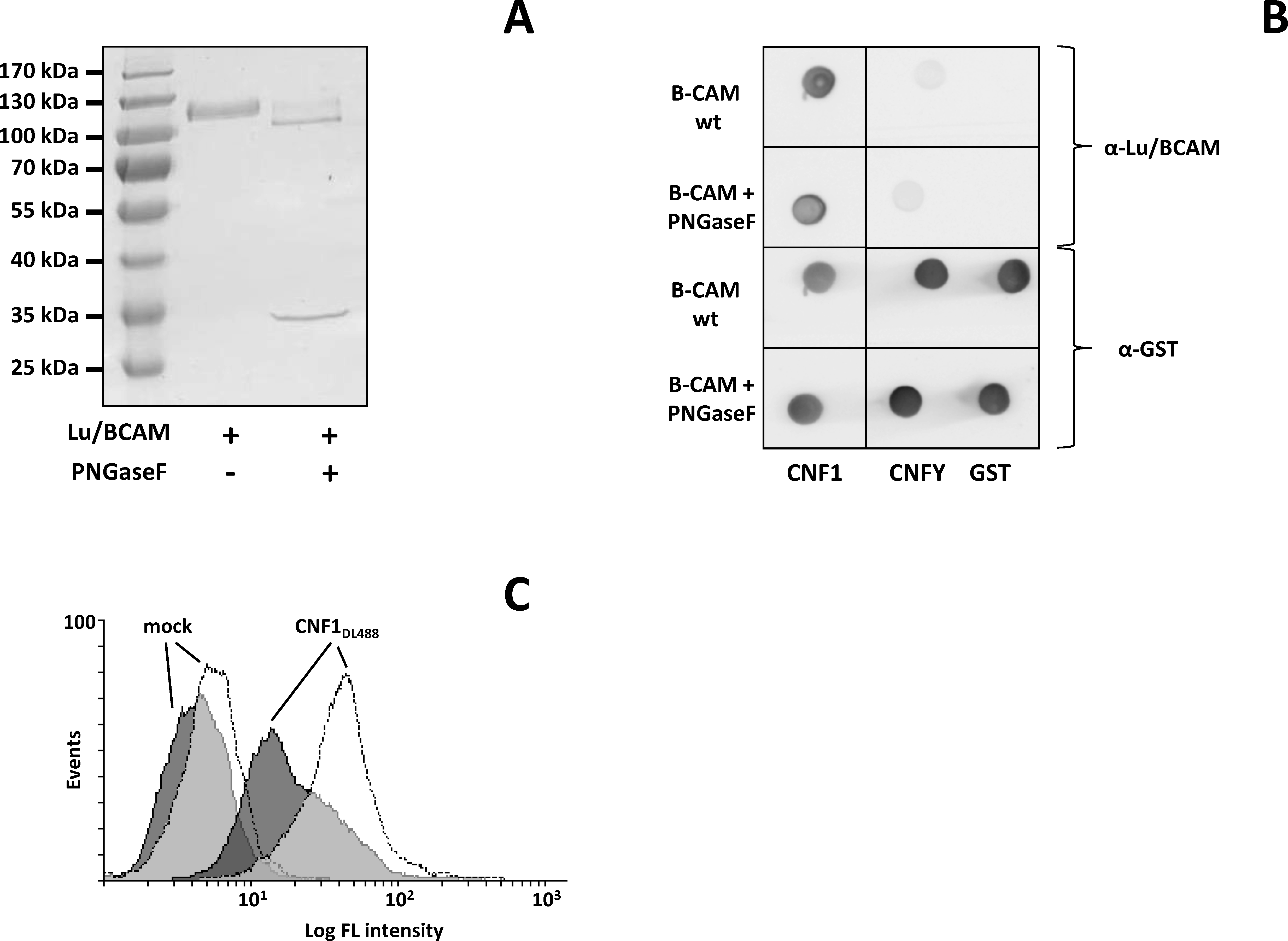

Supplement: Figure S4 — Role of Lu/BCAM glycosylation. Recombinant BCAM was treated with PNGaseF and analyzed deglycosylation by SDS-PAGE. De-glycosylated rBCAM runs faster according to its lower molecular weight (67 kDa) as compared with the glycosylated BCAM (84 kDa) (A). GST-CNF1, GST-CNFY and GST were spotted onto a nitrocellulose membrane. An overlay assay with glycosylated recombinant BCAM and PNGaseF-treated, de-glycosylated rBCAM was performed. Following washing bound rBCAM was detected with an anti-Lu/BCAM antibody. Equal protein load was analyzed by visualizing the GST part of the spotted proteins with an anti GST-antibody (B). Facs-analysis revealed that the toxin binds with higher affinity to the cells (C): Suspensions of PNGase F-treated (white, dashed lined peaks) or untreated (dark grey peaks) HEK293 cells (1×10^5^ cells in 1 ml medium) were incubated for 20 min at 4°C with 2 µg of DyLight488-labeled GST-CNF1 (CNF1DL488) or without protein (mock), washed with PBS, and subjected to FACS analysis. Results are presented as histogram plots, where single cell events are plotted against cell surface-bound fluorescence (Log FL intensity). (TIFF) [file ppat.1003884.s004.tiff]

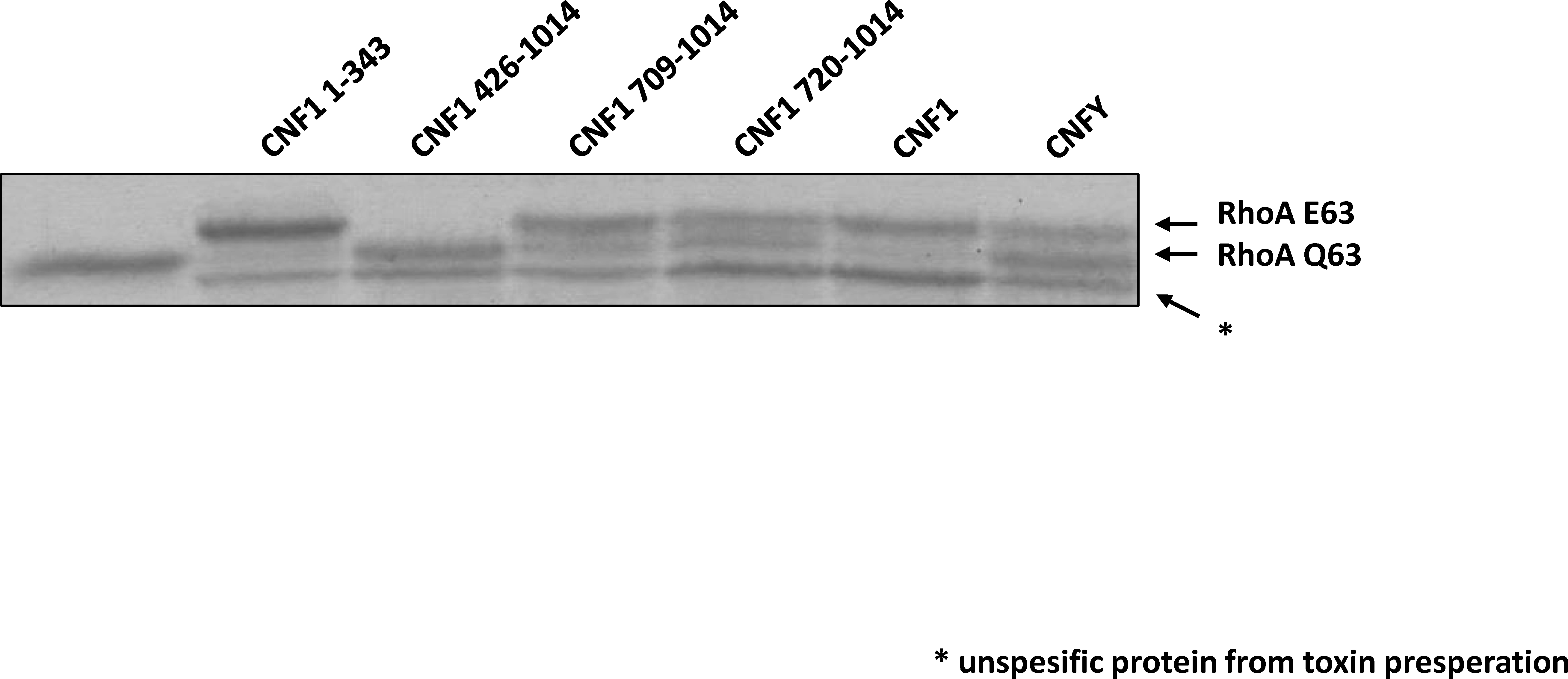

Supplement: Figure S5 — Recombinant CNF1 fragments are folded correctly. Recombinant RhoA (5 µM) was incubated with GST-CNF1 and GST-CNF1 fragments (each 1 µM), respectively as indicated in a buffer, containing 50 mM TRIS-HCl, pH 7.5, 5 mM MgCl2, 1 mM EDTA, and 1 mM DTT for 4 h at 37°C. Proteins were loaded onto 12.5% SDS-gel containing 1 M urea. The samples were analyzed for the typical shift of deamidated RhoA to higher molecular weight. (TIFF) [file ppat.1003884.s005.tiff]
